# Prognostic Value of Admission Mean Corpuscular Volume for Major Adverse Cardiovascular Events following Stent Implantation in Nondiabetic and Diabetic Patients with Acute Coronary Syndrome

**DOI:** 10.1155/2020/7054596

**Published:** 2020-07-17

**Authors:** Lele Cheng, Lisha Zhang, Junhui Liu, Wenyuan Li, Xiaofang Bai, Ruifeng Li, Bolin Li, Lijun Wang, Juan Zhou, Yue Wu, Zuyi Yuan

**Affiliations:** ^1^Department of Cardiovascular Medicine, The First Affiliated Hospital, Xi'an Jiaotong University, Xi'an, Shaanxi, China; ^2^Department of Clinical Laboratory, The First Affiliated Hospital, Xi'an Jiaotong University, Xi'an, Shaanxi, China; ^3^Key Laboratory of Molecular Cardiology, Shaanxi Province, Xi'an, Shaanxi, China; ^4^Key Laboratory of Environment and Genes Related to Diseases, Ministry of Education, Xi'an, Shaanxi, China

## Abstract

**Background:**

One of the key concerns of the clinician is to identify and manage risk factors for major adverse cardiovascular events (MACEs) in nondiabetic and diabetic patients with acute coronary syndrome (ACS) undergoing stent implantation. Mean corpuscular volume (MCV) is a marker of erythrocyte size and activity and is associated with prognosis of cardiovascular disease. However, the role of admission MCV in predicting MACEs following stent implantation in diabetes mellitus (DM), non-DM, or whole patients with ACS remains largely unknown.

**Methods and Results:**

A total of 437 ACS patients undergoing stent implantation, including 294 non-DM (59.08 ± 10.24 years) and 143 DM (63.02 ± 9.92 years), were analyzed. Admission MCV was higher in non-DM than DM patients. During a median of 31.93 months follow-up, Kaplan-Meier curve demonstrated that higher admission MCV level was significantly associated with increased MACEs in whole and non-DM, but not in DM patients. In Cox regression analysis, the highest MCV tertile was associated with higher MACEs in whole ([HR] 1.870, 95% CI 1.113-3.144, *P* = 0.018), especially those non-DM ([HR] 2.089, 95% CI 1.077-4.501, *P* = 0.029) patients after adjustment of several cardiovascular risk factors. MCV did not predict MACEs in DM patients. During landmark analysis, admission MCV showed better predictive value for MACEs in the first 32 months of follow-up than in the subsequent period. Finally, the receiver operating characteristic (ROC) curve was conducted to confirmed the value of admission MCV within 32 months.

**Conclusion:**

In patients with ACS, elevated admission MCV is an important and independent predictor for MACEs following stent implantation, especially amongst those without DM even after adjusting for lifestyle and clinical risk factors. However, as the follow-up period increased, the admission MCV lost its ability to predict MACEs.

## 1. Introduction

Coronary artery disease (CAD) is caused by atherosclerosis accompanied by stenosis or lumen obstruction, resulting in myocardial ischemia, hypoxia, and necrosis [1–3]. It has become an important threat to human life and health, especially in middle- and low-income countries [4, 5]. Acute coronary syndrome (ACS) is a serious type of CAD that can lead to various adverse outcomes [6, 7]. Percutaneous coronary intervention (PCI) is a common revascularization strategy in the management of ACS due to its safety profile, low complication rates, symptom relief, and survival benefits [8, 9]. Although significant achievements have been made in the treatment, major adverse cardiovascular events (MACEs) remains a frequent and severe complication following stent implantation [10, 11].

The incidence of diabetes mellitus (DM), a metabolic disorder characterized by hyperglycemia, is a major and important risk factor for cardiovascular events including ACS undergoing PCI [12, 13]. Sustained hyperglycemia is shown to exacerbate cardiac damage through changes of erythrocyte [14]. Mean corpuscular volume (MCV) is the measure of the average size of erythrocytes, which is closely related to erythrocyte disorders [15, 16]. It is reduced in diabetes, probably because iron deficiency anemia is common in people with diabetes [17, 18]. Studies demonstrate that MCV is associated with the prognosis of several diseases, including peripheral arterial disease, CAD, and cerebral ischemic stroke [19–23]. The possible mechanism is that the increase of MCV impairs the antioxidant capacity of erythrocytes and decreases endothelial function, both of which promote the development of atherosclerosis [24]. In addition, high MCV would be related to a hindered flow of larger, less flexible erythrocytes through microcirculation, which might contribute to myocardial ischemia [25, 26]. However, the role and difference of MCV in predict MACEs between nondiabetic and diabetic populations with ACS undergoing PCI still largely unknown. Therefore, exploring the relationship between MCV level and adverse outcomes following stent implantation in nondiabetic and diabetic patients with ACS is of great clinical value.

In this context, the present study was performed to explore the value of admission MCV for prognosis of MACEs in ACS patients, with or without DM in China.

## 2. Materials and Methods

### 2.1. Study Population

The present study was carried out in the First Affiliated Hospital of Medical College of Xi'an Jiaotong University from January 2013 to February 2014. The inclusion criteria were confirmed admission diagnosis of ACS including ST-elevation myocardial infarction (STEMI), non-ST-segment elevation myocardial infarction (NSTEMI), and unstable angina pectoris (UA), successful treatment by stent implantation, without diabetic ketosis or nonketotic hyperosmolar coma. The exclusion criteria were patients with malignant tumors, severe renal and liver diseases, pregnant patients, patients with autoimmune diseases or blood disorders, severe cerebrovascular diseases, and prior history of surgical treatment within 2 weeks of this study, and data were not available for the MCV levels. Qualitative and quantitative coronary angiographic analyses were carried out according to standard methods. PCI was performed using standard techniques. All patients were given loading doses of aspirin (300 mg), clopidogrel (600 mg), and unfractionated heparin (100 U/kg) before coronary intervention. The treatment strategy, stenting techniques, and use of glycoprotein IIb/IIIa receptor inhibitors or intravascular ultrasound used at the discretion of the operator during the PCI procedures. Drug-eluting stents were used (Medtronic, Inc.). The success of PCI is defined as previously described [27, 28]. Written informed consent was obtained from all study participants, approved by ethic committee approval at the First Affiliated Hospital of Xi'an Jiaotong University.

The estimation of sample size was performed using PASS software (version 11.0) [29]. A sample size of 311 achieves 90% power to detect an effect size of 0.202 using a 2 degrees of freedom chi-square test with a significance level of 0.050.

### 2.2. Demographic and Clinical Data

Main demographic data, cardiovascular risk factors, and cardiovascular drugs received were obtained from medical records. A diagnosis of DM was based on the criteria by the American Diabetes Association [30]. Hypertension was defined as resting blood pressure ≥140/90 mmHg at two different visits, and having a history of hypertension or receiving hypertension drugs. Previous MI was based on a history of acute MI (AMI) or with signs of an infarction outside the area of the index infarction.

### 2.3. Biochemical Measurements

Peripheral blood was sampled from patients within 24 h of admission prior to stent implantation. Triglycerides (TG), low-density lipoprotein cholesterol (LDL), high-sensitivity C-reactive protein (hsCRP), creatinine, Hemoglobin A1c (HbA1c), and pro-B-type natriuretic peptide (pro-BNP) were assayed using the Cobas Integra automated chemistry analyzer (Roche Cobas Integra 400 Plus, Roche Diagnostics, USA). High-sensitivity C-reactive protein was assessed using enzyme-linked immunosorbent assay according to the manufacturer's instructions (EIA-3954, DRG International Inc., Springfield Township, USA). Hemoglobin (HGB), MCV, and platelet counts were measured using the Sysmex KX 4000i haematology analyzer (Sysmex Corporation, Kobe, Japan) by standard biochemical techniques. The patients were further divided into 3 groups according to tertiles of MCV, and the reference MCV interval of our laboratory was 82-100 fL. Echocardiographs were performed on admission by experienced echo cardiologists, and systolic function was expressed as the ejection fraction (EF), which was calculated using Simpson's method.

### 2.4. Outcomes and Follow-Up

MACEs were defined as composite endpoints, including all-cause death, nonfatal AMI, urgent coronary revascularization, UA, and stroke [31]. All patients were followed up by interview or telephone in our hospital, and the end of follow-up was the date of the first MACEs occurrence.

### 2.5. Statistical Analysis

The statistical analyses in this study were performed using SPSS for Windows 25.0 (SPSS Inc, Chicago, IL), R software (version 3.6.1), and EmpowerStats (http://www.empowerstats.com/). Continuous variables were expressed as mean ± SD or median (interquartile range (IQR)) according to different distributions, and in case of normal distribution as confirmed by the Kolmogorov-Smirnov test. Unpaired *t*-test or one-way ANOVA or Mann-Whitney *U* test was used to test the statistical significance of differences between the means or medians where appropriate. Categorical variables were expressed as frequency and proportions, and compared with the chi-square or Fisher exact test. Kaplan-Meier survival curves were constructed to assess the association of different MCV level groups with the occurrence of MACEs, and Cox regression models were constructed to calculate hazard ratios (HRs) and 95% confidence intervals (CIs) for MACEs. Landmark analyses were performed according to a landmark point of the 32 months. HRs and 95% CIs were calculated separately for events occurring up to 32 months and those over the 32nd month to the end of follow-up. Finally, the ROC curve was conducted.

All probability values were two-tailed. *P* < 0.05 was considered statistically significant.

## 3. Results

### 3.1. Clinical Characteristics of Patients with and without DM

**Figure 1:**
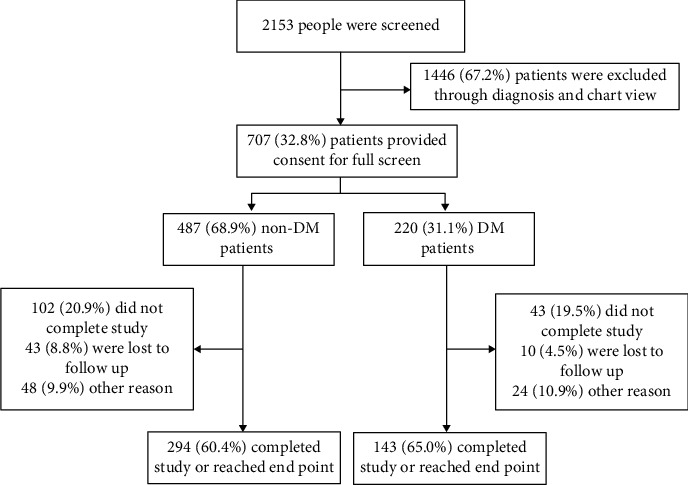
Study flow for the present analysis.

In the study, a total of 2153 patients were screened; 437 consecutive ACS patients who are undergoing stent implantation were analyzed, including 294 non-DM patients and 143 DM patients (Figure 1). During a median follow-up of 31.93 months, 140 (32.03%) were experienced MACEs. The basic characteristics of the whole, nondiabetic, and diabetic patients are presented in Table 1. Diabetic patients were elder, had lower MCV and HGB level compared with nondiabetic patients. Medications usage including aspirin (99.1%), clopidogrel (97.3%), ACEI/ARB (91.1%), calcium channel blocker (21.7%), *β*-blockers (88.1%), and statin (98.2%) are listed in Table 1.

### 3.2. Comparison of Basic Characteristics Stratified by MCV Tertiles

According to the above results, we found that the admission MCV level varied greatly between nondiabetic and diabetic patients. Therefore, all patients were segregated into three groups by tertiles of admission MCV level, and a comparison of the clinical data of these groups is shown in Table 2. The proportions of diabetes and HbA1c level were decreased as MCV level increased (all *P* < 0.05). Moreover, the incidence of MACEs in the MCV-H group (41.8%) was higher than that in the MCV-M (25.5%) and MCV-L group (28.8%, *P* = 0.007). No significant difference in other factors and medication use at discharge were observed amongst the three groups (all *P* > 0.05).

### 3.3. MACEs Occurrence

**Figure 2:**
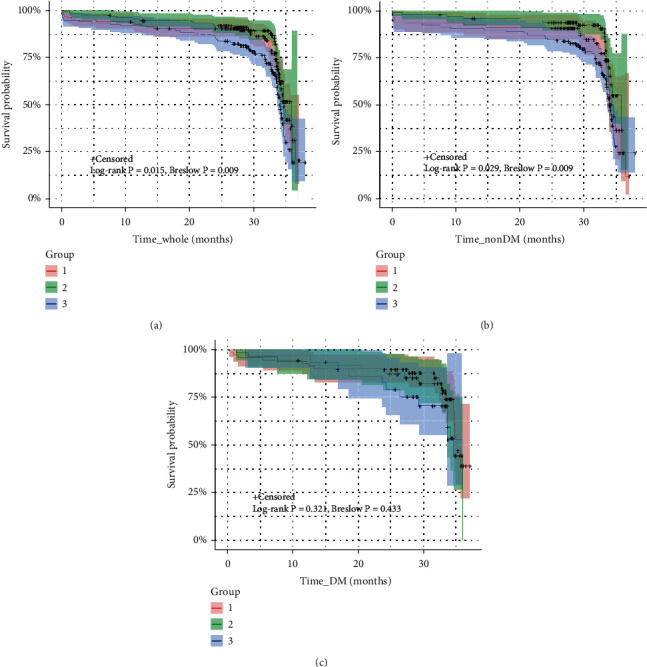
Kaplan-Meier survival curves for MACEs. Cumulative Kaplan-Meier survival curves for MACEs in the (a) whole ACS patients undergoing stent implantation (b) without DM and (c) with DM by MCV level. Group 1, MCV-L; Group 2, MCV-M; Group 3, MCV-H.

Kaplan-Meier survival analysis demonstrated that higher admission MCV level was significantly associated with MACEs occurrence both in whole (*P* = 0.015, log-rank test; *P* = 0.009, Breslow test) and non-DM patients (*P* = 0.029, log-rank test; *P* = 0.009, Breslow test), but not in DM patients (*P* = 0.321, log-rank test; *P* = 0.433, Breslow test), as shown in Figure 2.

We then used Cox regression model for further analysis as shown in Table 3. In univariate Cox regression analysis, we found MCV-H was significantly associated with an increased risk of MACEs in whole (HRs 1.540, 95% CI 1.038-2.284, *P* = 0.032) and non-DM patients (HRs 1.963, 95% CI 1.180-3.265, *P* = 0.009) over a median of 31.93 months of follow-up. This relationship remained significant in whole (HRs 1.870, 95% CI 1.113–3.144, *P* = 0.018), especially non-DM patients (HRs 2.089, 95% CI 1.077–4.501, *P* = 0.029) after adjustment for age, sex, body mass index, smoking, past myocardial infarction, hypertension, creatinine, creatine kinase isoenzymes, pro-B-type natriuretic peptide, and left ventricle ejection fraction. However, MCV level was not associated with the risk of MACEs in DM patients.

**Figure 3:**
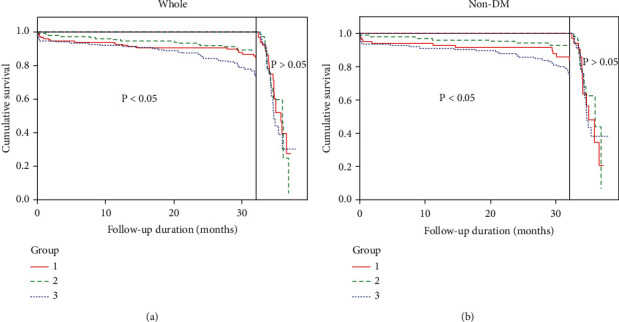
Landmark analysis of MACEs following stent implantation in whole and nondiabetic ACS patients. Kaplan-Meier survival curves (divided into two parts by the landmark point of 32 months) for MACEs (a) in the whole and (b) in non-DM population with ACS undergoing stent implantation by MCV level. Group 1, MCV-L; Group 2, MCV-M; Group 3, MCV-H.

**Figure 4:**
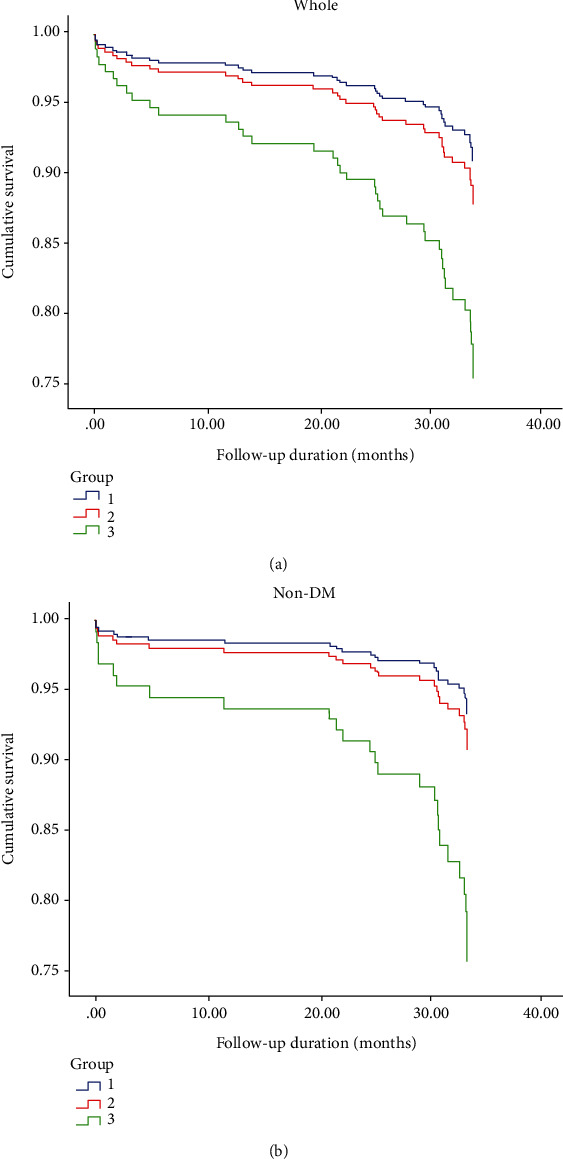
Cumulative survival curves for MACEs within 32 months. Cumulative survival curves for MACEs (a) in the whole and (b) non-DM population by MCV level within 32 months after adjust cardiovascular risk factors. Group 1, MCV-L; Group 2, MCV-M; Group 3, MCV-H.

Moreover, the whole and non-DM population were subjected to landmark survival analysis with a landmark point of 32 months. The cumulative survivals of MACEs were statistically different in whole and non-DM patients within 32 months, but with no significant differences in the time period over 32nd month to the end of follow-up (Figure 3). The results in Table 4 showed significantly higher MACEs in the MCV-H group than MCV-L group in the whole (HRs 2.979, 95% CI1.331-6.671, *P* = 0.020), especially non-DM population (HRs 4.054, 95% CI 1.192-13.789, *P* = 0.025) within 32 months of follow-up but not in the later period (over 32nd month to the end of follow-up). The corresponding graphs were shown in Figure 4.

**Figure 5:**
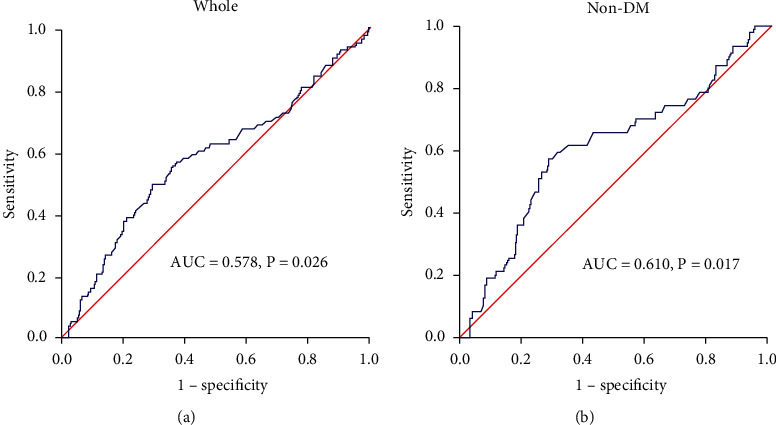
Receiver operating characteristic (ROC) curve analysis. ROC curve analysis on predictive value of admission MCV for MACEs following stent implantation (a) in whole ACS patients and (b) those without DM within 32 months.

To further evaluate the predictive value for MACEs of admission MCV within 32 months, we applied the ROC curve analysis. As shown in Figure 5 and Supplementary Tables 1–2, the areas under curve for MACEs was 0.578 (95% CI 0.506-0.651, *P* = 0.026) in whole and 0.610 (95% CI 0.517-0.703, *P* = 0.017) in non-DM patients, whereas there was no association between MCV and MACEs in DM patients (*P* > 0.05). The optimal cut-off threshold for admission MCV in predicting MACEs following stent implantation was 94.25 (sensitivity/specificity: 56.6%/62.7%) in whole and 95.95 (sensitivity/specificity: 57.4%/71.3%) in non-DM populations.

## 4. Discussion

Previous studies showed that there was the high risk of all-cause mortality in patients with ACS within one year after PCI [32]. Long-term glycometabolic disorder implied a high risk for cardiovascular disease and adverse outcomes [33, 34]. Consequently, it is urgent to identify and manage risk factors of nondiabetic and diabetic patients in order to ameliorate adverse cardiovascular events. In the current study, we noted that MCV levels were higher in nondiabetic than diabetic ACS patients following stent implantation. Intriguingly, Kaplan-Meier survival analysis and Cox regression analysis demonstrated that the incidence of MACEs following stent implantation was significantly higher in MCV-H group compared with MCV-L group of whole, especially those nondiabetic populations but not diabetic populations over a median of 31.93 months of follow-up. Moreover, landmark analysis indicated that, in comparison to the MCV-L group, the increased risk of MCV-H group within 32 months was lost after 32nd month to the end of follow-up. Finally, the prognostic value of admission MCV within 32 months was further confirmed by ROC curve analysis in whole and non-DM patients. To the best of our knowledge, this is the first study to focus on the relationship between admission MCV level and MACEs incidence following stent implantation in ACS patients with and without DM.

Chronic hyperglycemia induces ROS production, crosses the erythrocyte membrane, and oxidizes heme proteins, which have been shown to lead to the progressive loss of deformability of erythrocytes [35]. Diabetes can change hematological parameters that were related to hyperglycemia [14]. Previous studies observed that MCV was decreased in DM as compared to non-DM [17, 36]. Macrocytosis increased mortality, as well as main adverse cardiovascular and cerebrovascular events, among consecutive patients who underwent PCI [37]. Increased MCV is associated with a higher incidence of subsequent cardiovascular event or death in nonanemic AMI patients [25]. However, the level of admission MCV and its role in MACEs occurrence of diabetic and nondiabetic ACS patients undergoing stent implantation receives not much attention. In the present study, the admission MCV level of non-DM patients was higher than that of DM patients. In addition, patients with MCV-H values exhibited high risk of MACEs in whole and non-DM ACS patients but not DM patients. Our study not only confirms the relationship between MCV and DM but also further assesses the association of MCV with MACEs incidence following stent implantation in ACS patients with or without DM. However, the conflicting evidence regarding MCV level and cardiovascular outcomes is reported. Stable CAD patients undergoing PCI in the 1st quartile who had the lowest MCV values exhibited a high risk of restenosis [22]. Obviously, the target population of this study are participants with stable CAD, and their aim is to explore the association between MCV and incident restenosis; these details are very different from those of our study.

High MCV reduces the antioxidant function of red blood cells and is an independent prognosticator of impaired endothelial function measured via flow-mediated dilatation [24]. The direct mechanism of high MCV effect may be related to the increase of red blood cells resistance through microcirculation, which may lead to myocardial ischemia [25, 26]. These factors may also be involved in the high occurrence of MACEs of MCV-H group in ACS patients undergoing stent implantation in this study. It is noteworthy that the significant difference of MACEs only exists in whole and nondiabetic patients in the present study might be due to chronic hyperglycemia and high HbA1c leads to low MCV level in general in DM patients.

The important implication of the present study is that landmark analyses revealed a better predictive value of admission MCV for MACEs after stent implantation in the first 32 months than in the subsequent period, and ROC curve confirmed its predictive value within 32 months in whole, especially non-DM populations, suggesting that admission MCV level cannot accurately reflect patients' outcomes with prolonged follow-up. To identify high-risk patients, current guidelines recommend a standardized approach involving validated scoring systems such as the Global Registry of Acute Coronary Events (GRACE) score [38, 39], which provides validated prognostic information for MACEs in ACS patients [40, 41]. Therefore, combining admission MCV with the GRACE score may provide a more accurate risk estimation in ACS undergoing PCI which needs to be further explored.

The present study also has several limitations. Firstly, as an observational cohort study, there is inevitably some confounding bias, such as selection bias. Secondly, this research is a single-center study in a Chinese population, and the sample size is relatively small, especially in patients with DM; therefore, the comparisons of subgroups may lack power to detect significant differences for selected variables. Further multicenter and larger-scale surveys are required to confirm the results from this study and to elucidate the precise mechanisms. Moreover, the study only included ACS patients undergoing stent implantation, which suggests that the study results may not be extended to all ACS patients.

## 5. Conclusions

This study demonstrated that elevated admission MCV level was an important independent risk factor and had predictive value for MACEs following stent implantation in whole, especially those nondiabetic ACS patients. However, as the follow-up period increased, admission MCV lost its ability to predict MACEs. Measuring MCV, which is cheap and easy to obtain, might be helpful for a prognostic risk stratification of ACS patients undergoing stent implantation at a relatively short-term, especially those without DM.

## Figures and Tables

**Table 1:** Characteristics of whole ACS patients undergoing stent implantation and with or without DM.

Variable	Whole (*n* = 437)	Non-DM (*n* = 294)	DM (*n* = 143)	*P* value
Mean corpuscular volume, fl	93.30 (90.25-96.35)	94.05 (90.80-97.50)	91.30 (88.40-94.60)	<0.001
Age, years	60.35 ± 10.27	59.08 ± 10.24	63.02 ± 9.92	0.002
Sex				
Male, %	340 (77.8)	237 (80.6)	103 (72.3)	0.060
Body mass index, kg/m^2^	25.00 ± 3.26	25.05 ± 3.35	24.99 ± 3.23	0.558
Smoking, %	246 (56.3)	170 (57.8)	76 (53.1)	0.411
Past MI, %	67 (15.3)	50 (17.0)	17 (11.9)	0.211
Past PCI or CABG, %	85 (19.5)	59 (20.1)	26 (18.2)	0.735
Hypertension, %	227 (51.9)	148 (50.3)	79 (55.2)	0.389
Family history, %	176 (40.1)	125 (42.5)	51 (35.7)	0.205
Diastolic blood pressure, mmHg	80 (70-84)	80 (70-84)	80 (70-84)	0.934
Systolic blood pressure, mmHg	120 (114-140)	120 (112-140)	127 (114-140)	0.281
Heart rate, bmp	69 (61-78)	69 (61-78)	70 (62-77)	0.982
Hemoglobin A1c, %	6.00 (5.60-6.70)	5.70 (5.50-6.00)	7.40 (6.60-8.80)	<0.001
Left ventricle ejection fraction, %	61 (49-68)	62 (49-68)	59 (45-68)	0.117
Hemoglobin, g/L	140.00 (128.00-151.00)	141.00 (129.50-151.00)	137.00 (123.00-149.00)	0.032
Platelet, 10^3^ cells/dL	181.00 (147.00-217.00)	183.50 (149.70-224.00)	176.00 (140.00-205.00)	0.055
Creatinine, mg/dL	66.71 (58.27-77.37)	67.38 (58.66-77.40)	64.74 (56.04-77.37)	0.527
Triglycerides, mmol/L	1.42 (1.08-1.96)	1.42 (1.09-1.94)	1.42 (1.00-1.98)	0.854
High-density lipoprotein, mmol/L	0.87 (0.76-1.03)	0.89 (0.76-1.06)	0.84 (0.74-0.99)	0.134
Low-density lipoprotein, mmol/L	2.13 (1.63-2.68)	2.18 (1.65-2.73)	2.04 (1.56-2.59)	0.128
Apolipoprotein A1, g/L	1.05 (0.96-1.19)	1.05 (0.96-1.19)	1.05 (0.94-1.19	0.678
High-sensitivity C-reactive protein, mg/L	1.40 (0.74-3.50)	1.46 (0.76-3.61)	1.19 (0.64-2.99)	0.375
Creatine kinase isoenzymes MB, U/L	15.70 (11.44-32.33)	15.70 (11.30-35.60)	15.75 (12.10-26.50)	0.864
Pro-B-type natriuretic peptide, pg/mL	377.45 (122.86-1008.50)	377.45 (122.18-1003.50)	377.80 (135.86-1063.25)	0.924
Medicine at discharge, %				
Aspirin	433 (99.1)	291 (99.0)	142 (99.3)	a
Clopidogrel	425 (97.3)	285 (96.9)	140 (97.9)	0.679
ACEI/ARB	398 (91.1)	269 (91.5)	129 (90.2)	0.792
Calcium channel blocker	95 (21.7)	60 (20.4)	35 (24.5)	0.399
*β*-Blockers	385 (88.1)	262 (89.1)	123 (86.0)	0.434
Statin	429 (98.2)	289 (98.3)	140 (97.9)	a

Data are presented as mean ± SD, median (IQR) or number (%), “a” represents *P* value = 1. ACS: acute coronary syndrome; DM: diabetes mellitus; MI: myocardial infarction; PCI or CABG: past percutaneous coronary intervention or coronary artery bypass grafting; ACEI: angiotensin-converting enzyme inhibition; ARB: angiotensin receptor blocker.

**Table 2:** Clinical characteristics stratified by MCV tertiles.

Variable	Mean corpuscular volume, fl			
	MCV-L (64.6-91.0) *N* = 146	MCV-M (91.1-95.3) *N* = 145	MCV-H (95.4-113.3) *N* = 146	*P* value
Mean corpuscular volume, fl	88.6 (86.5-90.25)	93.30 (91.90-94.20)	98.00 (96.30-100.55)	<0.001
Age, year	59.24 ± 10.6	60.43 ± 9.7	61.36 ± 10.4	0.210
Sex				
Male, %	105 (74.0)	115 (79.3)	120 (80.1)	0.183
Body mass index, kg/m^2^	25.59 ± 3.09	24.78 ± 3.36	24.66 ± 3.26	0.064
Smoking, %	85 (58.2)	71 (49.0)	90 (65.8)	0.079
Past MI, %	23 (15.8)	23 (15.9)	22 (15.1)	0.980
Past PCI or CABG, %	31 (21.2)	30 (20.7)	25 (17.1)	0.631
Hypertension, %	84 (57.5)	75 (51.7)	69 (47.3)	0.212
Diabetes, %	67 (45.9)	47 (32.4)	29 (19.9)	<0.001
Family history, %	59 (40.4)	52 (35.9)	65 (44.5)	0.322
Diastolic blood pressure, mmHg	80 (70-90)	80 (70-85)	80 (70-86)	0.154
Systolic blood pressure, mmHg	120 (118-140)	120 (110-140)	125 (114-140)	0.811
Heart rate, bmp	70 (62-76)	69 (61-77)	67 (61-78)	0.764
Hemoglobin A1c, %	6.15 (5.70-7.65)	6.05 (5.62-6.50)	5.80 (5.50-6.20)	<0.001
Left ventricle ejection fraction, %	62 (48-69)	62 (47-68)	61 (49-68)	0.873
Hemoglobin, g/L	139.00 (125.50-150.50)	140.00 (130.00-152.00)	141.00 (127.00-151.00)	0.846
Platelet, 10^3^ cells/dL	186.00 (155.50-219.00)	181.00 (143.75-218.00)	172.00 (137.00-214.00)	0.230
Creatinine, mg/dL	66.43 (57.35-76.69)	66.32 (57.26-75.33)	78.81 (60.05-79.60)	0.174
Triglycerides, mmol/L	1.49 (1.11-2.06)	1.41 (1.09-1.96)	1.41 (0.98-1.80)	0.887
High-density lipoprotein, mmol/L	0.87 (0.77-1.03)	0.89 (0.76-1.28)	0.87 (0.74-1.07)	0.670
Low-density lipoprotein, mmol/L	2.14 (1.67-2.73)	2.19 (1.74-2.63)	2.02 (1.46-2.73)	0.336
High-sensitivity C-reactive protein, mg/L	1.53 (0.74-3.63)	1.16 (0.69-3.00)	1.38 (0.74-3.78)	0.423
Creatine kinase isoenzymes MB, U/L	14.20 (10.91-26.50)	16.85 (12.20-35.03)	16.75 (12.24-37.96)	0.068
Pro-B-type natriuretic peptide, pg/ML	368.90 (116.60-886.30)	403.80 (171.50-1005.00)	335.20 (121.4-1160.00)	0.724
Major adverse cardiovascular events, %	42 (28.8)	37 (25.5)	61 (41.8)	0.007
Type of acute coronary syndrome, %				0.781
Unstable angina	86 (58.9)	76 (52.4)	78 (53.4)	
ST-segment elevation myocardial infarction	40 (27.4)	46 (31.7)	48 (32.9)	
Non-ST-segment elevation myocardial infarction	20 (13.7)	23 (15.9)	20 (13.7)	
Lesion vessel number, %				0.083
1	15 (10.3)	27 (18.6)	26 (17.8)	
2	43 (29.5)	48 (33.1)	35 (24.0)	
3	88 (60.3)	70 (48.3)	85 (58.2)	
Medicine at discharge, %				
Aspirin	146 (100)	145 (100)	142 (97.3)	a
Clopidogrel	140 (95.9)	143 (98.6)	142 (97.3)	0.404
ACEI/ARB	133 (91.1)	134 (92.4)	131 (89.7)	0.724
Calcium channel blockers	40 (27.4)	24 (16.4)	31 (21.2)	0.080
*β*-Blockers	125 (85.6)	126 (86.9)	134 (91.8)	0.230
Statin	143 (97.9)	143 (98.6)	143 (97.9)	a

Data are presented as mean ± SD, median (IQR) or number (%); “a” represents *P* value =1. MCV: mean corpuscular volume; MI: myocardial infarction; PCI or CABG: past percutaneous coronary intervention or coronary artery bypass grafting; ACEI: angiotensin-converting enzyme inhibition; ARB: angiotensin receptor blocker.

**Table 3:** Cox regression analysis of MACEs in whole, nondiabetic, and diabetic patients.

Variable	Univariate analysis			Multivariate analysis		
	Hazard ratio (95% CI)	SEM	*P* value	Hazard ratio (95% CI)	SEM	*P* value
Whole						
MCV-L	Reference		0.032	Reference		0.032
MCV-M^b^	0.899 (0.577-1.401)	0.226	0.638	1.159 (0.662-2.028)	0.286	0.606
MCV-H^b^	1.540 (1.038-2.284)	0.201	0.032	1.870 (1.113-3.144)	0.265	0.018
Non-DM						
MCV-L	Reference		0.032	Reference		0.032
MCV-M^b^	1.487 (0.827-2.673)	0.299	0.185	1.223 (0.574-2.606)	0.386	0.415
MCV-H^b^	1.963 (1.180-3.265)	0.260	0.009	2.089 (1.077-4.501)	0.338	0.029
DM						
MCV-L	Reference		0.328			
MCV-M^b^	1.368 (0.695-2.692)	0.345	0.364			
MCV-H^b^	1.795 (0.816-3.950)	0.402	0.146			

MACEs: major adverse cardiovascular events; MCV: mean corpuscular volume; DM, diabetes mellitus; CI: confidence interval; SEM: standard error of measurement. Adjusted for age, sex, body mass index, smoking, past myocardial infarction, hypertension, creatinine, creatine kinase isoenzymes, pro-B-type natriuretic peptide, and left ventricle ejection fraction. ^b^Compared with MCV-L group.

**Table 4:** Landmark analysis of MACEs in whole and nondiabetic patients.

Variable	Hazard ratio (95% CI)	SEM	*P* value
Whole (≤32 months)			
MCV-L	Reference		
MCV-M^b^	1.359 (0.536-3.447)	0.478	0.518
MCV-H^b^	2.979 (1.331-6.671)	0.411	0.020
Whole (>32 months to maximum follow-up)			
MCV-L	Reference		
MCV-M^b^	0.933 (0.447-1.945)	0.375	0.852
MCV-H^b^	1.410 (0.675-2.948)	0.376	0.361
Non-DM (≤32 months)			
MCV-L	Reference		
MCV-M^b^	1.400 (0.334-5.864)	0.731	0.645
MCV-H^b^	4.054 (1.192-13.789)	0.625	0.025
Non-DM (>32 months to maximum follow-up)			
MCV-L	Reference		
MCV-M^b^	1.119 (0.420-2.985)	0.501	0.822
MCV-H^b^	1.596 (0.647-3.936)	0.461	0.310

ACS: acute coronary syndrome; MACEs: major adverse cardiovascular events; MCV: mean corpuscular volume; CI: confidence interval; SEM: standard error of measurement. ^b^Compared with MCV-L group.

## Data Availability

The datasets analyzed during the current study are available from the corresponding author on reasonable request.

## Abbreviations

MCV: Mean corpuscular volume
ACS: Acute coronary syndromes
PCI: Percutaneous coronary intervention
DM: Diabetes mellitus
HbA1c: Hemoglobin A1c
CAD: Coronary artery disease
AMI: Acute myocardial infarction
UA: Unstable angina
STEMI: ST-segment elevation myocardial infarction
NSTEMI: Non-ST-segment elevation myocardial infarction
MACEs: Major adverse cardiovascular events
CABG: Percutaneous coronary intervention or coronary artery bypass grafting
HGB: Hemoglobin
hsCRP: High-sensitivity C-reactive protein
CKMB: Creatine kinase isoenzymes MB
pro-BNP: Pro-B-type natriuretic peptide
EF: Ejection fraction
TG: Triglyceride
LDL: Low-density lipoprotein cholesterol
HDL: High-density lipoprotein cholesterol
ACEI: Angiotensin-converting enzyme inhibition
ARB: Angiotensin receptor blocker
CCB: Calcium channel blocker
GRACE: Global registry of acute coronary events.

## Supplementary Materials

Supplementary Materials Supplementary Table 1: the coordinate of ROC Curve for admission MCV associated with MACEs in non-DM populations within 32 months. Supplementary Table 2: the coordinate of ROC Curve for admission MCV associated with MACEs in whole populations within 32 months.
