# Supplementary material for: Prognostic Value of Admission Mean Corpuscular Volume for Major Adverse Cardiovascular Events following Stent Implantation in Nondiabetic and Diabetic Patients with Acute Coronary Syndrome
Source: Dis Markers. 2020 Jul 17;2020:7054596. doi: 10.1155/2020/7054596 (PMC7383318; doi:10.1155/2020/7054596)
Supplement: Supplementary Materials — Supplementary Table 1: the coordinate of ROC Curve for admission MCV associated with MACEs in non-DM populations within 32 months. Supplementary Table 2: the coordinate of ROC Curve for admission MCV associated with MACEs in whole populations within 32 months. [file 7054596.f1.docx]

Supplementary Table 1 The coordinate of ROC Curve for admission MCV associated with MACEs in non-DM populations within 32 months

|  | **Sensitivity** | 1 - **Specificity** |
| --- | --- | --- |
| 63.599999999999994 | 1.000 | 1.000 |
| 69.699999999999990 | 1.000 | .996 |
| 78.600000000000000 | 1.000 | .992 |
| 82.700000000000000 | 1.000 | .988 |
| 83.500000000000000 | 1.000 | .984 |
| 84.150000000000000 | 1.000 | .980 |
| 84.850000000000000 | 1.000 | .976 |
| 85.450000000000000 | 1.000 | .972 |
| 85.700000000000000 | 1.000 | .968 |
| 85.950000000000000 | 1.000 | .951 |
| 86.100000000000000 | 1.000 | .947 |
| 86.300000000000010 | .979 | .943 |
| 86.450000000000000 | .979 | .939 |
| 86.600000000000000 | .979 | .935 |
| 86.850000000000000 | .979 | .931 |
| 87.050000000000000 | .957 | .931 |
| 87.150000000000000 | .957 | .927 |
| 87.250000000000000 | .936 | .923 |
| 87.449999999999990 | .936 | .919 |
| 87.650000000000000 | .936 | .911 |
| 87.800000000000010 | .936 | .907 |
| 87.950000000000000 | .936 | .903 |
| 88.050000000000000 | .936 | .899 |
| 88.150000000000000 | .936 | .887 |
| 88.250000000000000 | .936 | .883 |
| 88.350000000000000 | .936 | .879 |
| 88.450000000000000 | .915 | .879 |
| 88.600000000000000 | .915 | .874 |
| 88.800000000000010 | .915 | .870 |
| 88.950000000000000 | .894 | .862 |
| 89.050000000000000 | .894 | .858 |
| 89.150000000000000 | .872 | .858 |
| 89.250000000000000 | .872 | .846 |
| 89.350000000000000 | .872 | .838 |
| 89.450000000000000 | .872 | .834 |
| 89.600000000000000 | .872 | .830 |
| 89.800000000000010 | .872 | .822 |
| 89.950000000000000 | .851 | .822 |
| 90.050000000000000 | .830 | .818 |
| 90.150000000000000 | .830 | .814 |
| 90.250000000000000 | .809 | .802 |
| 90.350000000000000 | .787 | .798 |
| 90.450000000000000 | .787 | .794 |
| 90.550000000000000 | .787 | .769 |
| 90.699999999999990 | .766 | .757 |
| 90.850000000000000 | .766 | .741 |
| 90.950000000000000 | .766 | .737 |
| 91.050000000000000 | .766 | .733 |
| 91.150000000000000 | .745 | .721 |
| 91.250000000000000 | .745 | .717 |
| 91.350000000000000 | .745 | .713 |
| 91.450000000000000 | .745 | .700 |
| 91.550000000000000 | .745 | .684 |
| 91.650000000000000 | .745 | .680 |
| 91.750000000000000 | .745 | .668 |
| 91.850000000000000 | .745 | .660 |
| 91.950000000000000 | .723 | .648 |
| 92.050000000000000 | .723 | .644 |
| 92.199999999999990 | .723 | .636 |
| 92.350000000000000 | .723 | .632 |
| 92.450000000000000 | .702 | .628 |
| 92.600000000000000 | .702 | .623 |
| 92.750000000000000 | .702 | .611 |
| 92.850000000000000 | .702 | .607 |
| 92.950000000000000 | .702 | .587 |
| 93.050000000000000 | .702 | .575 |
| 93.150000000000000 | .702 | .567 |
| 93.250000000000000 | .681 | .563 |
| 93.350000000000000 | .681 | .551 |
| 93.450000000000000 | .660 | .538 |
| 93.550000000000000 | .660 | .518 |
| 93.650000000000000 | .660 | .514 |
| 93.750000000000000 | .660 | .502 |
| 93.850000000000000 | .660 | .490 |
| 93.950000000000000 | .660 | .474 |
| 94.050000000000000 | .660 | .470 |
| 94.150000000000000 | .660 | .453 |
| 94.250000000000000 | .660 | .441 |
| 94.350000000000000 | .660 | .433 |
| 94.500000000000000 | .660 | .429 |
| 94.650000000000000 | .638 | .421 |
| 94.800000000000010 | .617 | .409 |
| 94.950000000000000 | .617 | .401 |
| 95.050000000000000 | .617 | .381 |
| 95.150000000000000 | .617 | .368 |
| 95.250000000000000 | .617 | .360 |
| 95.350000000000000 | .617 | .356 |
| 95.450000000000000 | .617 | .352 |
| 95.550000000000000 | .617 | .348 |
| 95.650000000000000 | .596 | .324 |
| 95.750000000000000 | .596 | .316 |
| 95.850000000000000 | .574 | .300 |
| 95.950000000000000 | .574 | .287 |
| 96.100000000000000 | .532 | .279 |
| 96.250000000000000 | .532 | .275 |
| 96.350000000000000 | .532 | .267 |
| 96.500000000000000 | .532 | .263 |
| 96.650000000000000 | .511 | .263 |
| 96.750000000000000 | .511 | .259 |
| 96.900000000000000 | .511 | .255 |
| 97.050000000000000 | .468 | .255 |
| 97.150000000000000 | .468 | .247 |
| 97.250000000000000 | .468 | .243 |
| 97.350000000000000 | .447 | .231 |
| 97.450000000000000 | .404 | .223 |
| 97.550000000000000 | .383 | .206 |
| 97.650000000000000 | .362 | .206 |
| 97.800000000000010 | .362 | .194 |
| 98.050000000000010 | .362 | .186 |
| 98.250000000000000 | .298 | .178 |
| 98.350000000000000 | .277 | .178 |
| 98.450000000000000 | .255 | .174 |
| 98.550000000000000 | .255 | .170 |
| 98.699999999999990 | .255 | .166 |
| 98.850000000000000 | .255 | .158 |
| 98.950000000000000 | .234 | .150 |
| 99.050000000000000 | .234 | .146 |
| 99.150000000000000 | .213 | .142 |
| 99.250000000000000 | .213 | .138 |
| 99.350000000000000 | .213 | .126 |
| 99.450000000000000 | .213 | .121 |
| 99.650000000000000 | .213 | .117 |
| 99.900000000000000 | .191 | .113 |
| 100.050000000000000 | .191 | .105 |
| 100.250000000000000 | .191 | .101 |
| 100.450000000000000 | .191 | .097 |
| 100.600000000000000 | .191 | .093 |
| 100.750000000000000 | .191 | .089 |
| 100.850000000000000 | .170 | .085 |
| 101.000000000000000 | .170 | .081 |
| 101.199999999999990 | .128 | .081 |
| 101.449999999999990 | .128 | .077 |
| 101.750000000000000 | .106 | .077 |
| 102.150000000000000 | .085 | .069 |
| 102.450000000000000 | .085 | .065 |
| 102.550000000000000 | .085 | .061 |
| 102.650000000000000 | .085 | .057 |
| 102.750000000000000 | .085 | .053 |
| 102.850000000000000 | .085 | .045 |
| 102.950000000000000 | .064 | .040 |
| 103.300000000000000 | .064 | .032 |
| 104.000000000000000 | .043 | .032 |
| 104.500000000000000 | .021 | .032 |
| 105.199999999999990 | .000 | .032 |
| 106.050000000000000 | .000 | .028 |
| 107.250000000000000 | .000 | .024 |
| 108.600000000000000 | .000 | .020 |
| 109.150000000000000 | .000 | .016 |
| 109.500000000000000 | .000 | .012 |
| 111.300000000000010 | .000 | .008 |
| 113.100000000000000 | .000 | .004 |
| 114.300000000000000 | .000 | .000 |

Supplementary Table 2 The coordinate of ROC Curve for admission MCV associated with MACEs in whole populations within 32 months

|  | **Sensitivity** | 1 - **Specificity** |
| --- | --- | --- |
| 63.599999999999994 | 1.000 | 1.000 |
| 69.699999999999990 | 1.000 | .997 |
| 75.199999999999990 | 1.000 | .994 |
| 78.250000000000000 | .988 | .994 |
| 81.650000000000000 | .988 | .992 |
| 82.600000000000000 | .976 | .992 |
| 82.900000000000000 | .976 | .986 |
| 83.500000000000000 | .976 | .983 |
| 84.100000000000000 | .976 | .980 |
| 84.250000000000000 | .964 | .980 |
| 84.350000000000000 | .964 | .977 |
| 84.550000000000010 | .964 | .972 |
| 84.750000000000000 | .952 | .972 |
| 84.900000000000000 | .952 | .969 |
| 85.050000000000000 | .952 | .966 |
| 85.150000000000000 | .952 | .963 |
| 85.250000000000000 | .952 | .958 |
| 85.350000000000000 | .952 | .955 |
| 85.450000000000000 | .940 | .952 |
| 85.600000000000000 | .940 | .949 |
| 85.750000000000000 | .940 | .946 |
| 85.850000000000000 | .940 | .944 |
| 85.950000000000000 | .940 | .932 |
| 86.050000000000000 | .940 | .929 |
| 86.150000000000000 | .940 | .927 |
| 86.300000000000010 | .928 | .924 |
| 86.450000000000000 | .928 | .921 |
| 86.550000000000000 | .928 | .912 |
| 86.650000000000000 | .928 | .910 |
| 86.750000000000000 | .928 | .907 |
| 86.900000000000000 | .928 | .904 |
| 87.050000000000000 | .916 | .901 |
| 87.150000000000000 | .916 | .895 |
| 87.250000000000000 | .904 | .893 |
| 87.400000000000000 | .904 | .881 |
| 87.550000000000000 | .892 | .881 |
| 87.650000000000000 | .880 | .876 |
| 87.800000000000010 | .880 | .873 |
| 87.950000000000000 | .880 | .870 |
| 88.050000000000000 | .880 | .864 |
| 88.150000000000000 | .880 | .856 |
| 88.250000000000000 | .867 | .847 |
| 88.350000000000000 | .855 | .842 |
| 88.450000000000000 | .843 | .839 |
| 88.550000000000000 | .843 | .833 |
| 88.650000000000000 | .843 | .828 |
| 88.750000000000000 | .843 | .822 |
| 88.850000000000000 | .843 | .819 |
| 88.950000000000000 | .819 | .816 |
| 89.050000000000000 | .819 | .814 |
| 89.150000000000000 | .807 | .811 |
| 89.250000000000000 | .807 | .802 |
| 89.350000000000000 | .807 | .791 |
| 89.450000000000000 | .807 | .788 |
| 89.600000000000000 | .807 | .782 |
| 89.750000000000000 | .807 | .777 |
| 89.850000000000000 | .795 | .774 |
| 89.950000000000000 | .783 | .768 |
| 90.050000000000000 | .771 | .763 |
| 90.150000000000000 | .771 | .757 |
| 90.250000000000000 | .759 | .749 |
| 90.350000000000000 | .735 | .743 |
| 90.450000000000000 | .723 | .734 |
| 90.550000000000000 | .723 | .715 |
| 90.650000000000000 | .711 | .701 |
| 90.750000000000000 | .711 | .695 |
| 90.850000000000000 | .699 | .678 |
| 90.950000000000000 | .699 | .675 |
| 91.050000000000000 | .699 | .664 |
| 91.150000000000000 | .687 | .650 |
| 91.250000000000000 | .687 | .644 |
| 91.350000000000000 | .687 | .636 |
| 91.450000000000000 | .675 | .624 |
| 91.550000000000000 | .675 | .607 |
| 91.650000000000000 | .675 | .599 |
| 91.750000000000000 | .675 | .588 |
| 91.850000000000000 | .663 | .576 |
| 91.950000000000000 | .639 | .565 |
| 92.050000000000000 | .639 | .556 |
| 92.199999999999990 | .639 | .548 |
| 92.350000000000000 | .639 | .545 |
| 92.450000000000000 | .627 | .542 |
| 92.600000000000000 | .627 | .537 |
| 92.750000000000000 | .627 | .528 |
| 92.850000000000000 | .627 | .525 |
| 92.950000000000000 | .627 | .508 |
| 93.050000000000000 | .627 | .494 |
| 93.150000000000000 | .627 | .483 |
| 93.250000000000000 | .614 | .477 |
| 93.350000000000000 | .614 | .469 |
| 93.450000000000000 | .602 | .460 |
| 93.550000000000000 | .602 | .444 |
| 93.650000000000000 | .590 | .435 |
| 93.750000000000000 | .590 | .424 |
| 93.850000000000000 | .578 | .410 |
| 93.950000000000000 | .578 | .398 |
| 94.050000000000000 | .578 | .395 |
| 94.150000000000000 | .566 | .384 |
| 94.250000000000000 | .566 | .373 |
| 94.350000000000000 | .554 | .367 |
| 94.500000000000000 | .554 | .359 |
| 94.650000000000000 | .530 | .350 |
| 94.800000000000010 | .518 | .339 |
| 94.950000000000000 | .494 | .333 |
| 95.050000000000000 | .494 | .316 |
| 95.150000000000000 | .494 | .308 |
| 95.250000000000000 | .494 | .299 |
| 95.350000000000000 | .494 | .297 |
| 95.450000000000000 | .482 | .294 |
| 95.550000000000000 | .482 | .291 |
| 95.650000000000000 | .434 | .277 |
| 95.750000000000000 | .434 | .268 |
| 95.850000000000000 | .422 | .254 |
| 95.950000000000000 | .410 | .240 |
| 96.050000000000000 | .386 | .232 |
| 96.150000000000000 | .386 | .226 |
| 96.250000000000000 | .386 | .223 |
| 96.350000000000000 | .386 | .218 |
| 96.500000000000000 | .386 | .212 |
| 96.650000000000000 | .373 | .212 |
| 96.750000000000000 | .373 | .206 |
| 96.900000000000000 | .373 | .203 |
| 97.050000000000000 | .349 | .201 |
| 97.150000000000000 | .325 | .192 |
| 97.250000000000000 | .325 | .189 |
| 97.350000000000000 | .313 | .181 |
| 97.450000000000000 | .289 | .175 |
| 97.550000000000000 | .277 | .164 |
| 97.650000000000000 | .265 | .161 |
| 97.800000000000010 | .265 | .150 |
| 98.000000000000000 | .265 | .144 |
| 98.150000000000000 | .265 | .141 |
| 98.250000000000000 | .229 | .136 |
| 98.350000000000000 | .217 | .136 |
| 98.450000000000000 | .205 | .130 |
| 98.550000000000000 | .205 | .124 |
| 98.699999999999990 | .205 | .121 |
| 98.850000000000000 | .205 | .116 |
| 98.950000000000000 | .181 | .113 |
| 99.050000000000000 | .181 | .110 |
| 99.150000000000000 | .169 | .107 |
| 99.250000000000000 | .157 | .107 |
| 99.350000000000000 | .157 | .096 |
| 99.450000000000000 | .145 | .093 |
| 99.600000000000000 | .145 | .090 |
| 99.750000000000000 | .145 | .088 |
| 99.900000000000000 | .133 | .085 |
| 100.050000000000000 | .133 | .079 |
| 100.250000000000000 | .133 | .076 |
| 100.450000000000000 | .133 | .073 |
| 100.600000000000000 | .133 | .071 |
| 100.750000000000000 | .133 | .068 |
| 100.850000000000000 | .120 | .065 |
| 101.000000000000000 | .120 | .062 |
| 101.150000000000000 | .084 | .062 |
| 101.250000000000000 | .084 | .059 |
| 101.449999999999990 | .084 | .056 |
| 101.750000000000000 | .072 | .056 |
| 101.950000000000000 | .060 | .051 |
| 102.100000000000000 | .048 | .051 |
| 102.300000000000010 | .048 | .048 |
| 102.450000000000000 | .048 | .045 |
| 102.550000000000000 | .048 | .042 |
| 102.650000000000000 | .048 | .040 |
| 102.750000000000000 | .048 | .037 |
| 102.850000000000000 | .048 | .031 |
| 102.950000000000000 | .036 | .028 |
| 103.300000000000000 | .036 | .023 |
| 104.000000000000000 | .024 | .023 |
| 104.500000000000000 | .012 | .023 |
| 105.199999999999990 | .000 | .023 |
| 106.050000000000000 | .000 | .020 |
| 107.250000000000000 | .000 | .017 |
| 108.600000000000000 | .000 | .014 |
| 109.150000000000000 | .000 | .011 |
| 109.500000000000000 | .000 | .008 |
| 111.300000000000010 | .000 | .006 |
| 113.100000000000000 | .000 | .003 |
| 114.300000000000000 | .000 | .000 |
